# A Programmable Plug & Play Sensor Interface for WSN Applications

**DOI:** 10.3390/s110909009

**Published:** 2011-09-21

**Authors:** Sergio D. Vera, Alberto Bayo, Nicolás Medrano, Belén Calvo, Santiago Celma

**Affiliations:** Group of Electronic Design, Aragon Institute for Engineering Research, I3A, Facultad de Ciencias, Pedro Cerbuna 12, 50009 Zaragoza, Spain; E-Mails: svera@unizar.es (S.D.V.); bayo@unizar.es (A.B.); becalvo@unizar.es (B.C.); scelma@unizar.es (S.C.)

**Keywords:** embedded microcontroller, plug & play, sensor interface, smart sensors, TEDS, wireless sensor networks

## Abstract

Cost reduction in wireless sensor networks (WSN) becomes a priority when extending their application to fields where a great number of sensors is needed, such as habitat monitoring, precision agriculture or diffuse greenhouse emission measurement. In these cases, the use of smart sensors is expensive, consequently requiring the use of low-cost sensors. The solution to convert such generic low-cost sensors into intelligent ones leads to the implementation of a versatile system with enhanced processing and storage capabilities to attain a plug and play electronic interface able to adapt to all the sensors used. This paper focuses on this issue and presents a low-voltage plug & play reprogrammable interface capable of adapting to different sensor types and achieving an optimum reading performance for every sensor. The proposed interface, which includes both electronic and software elements so that it can be easily integrated in WSN nodes, is described and experimental test results to validate its performance are given.

## 1. Introduction

The ever-increasing reduction of sensor size has favored their integration in embedded sensing applications. This fact, together with the recent advances in mobile communications, has made it possible to use low-cost low-power sensor networks which interact in widely diverse environments by means of wireless communication protocols [1]. In this way, a broad range of innovative applications arises, such as environmental monitoring, military sensor networks, healthcare applications, networks for detecting chemical, biological, or radiological risks, traffic sensor networks, manufacturing automation, forest fire detection, *etc*.

Numerous applications of Wireless Sensor Networks (WSNs) involve monitoring physical and chemical parameters over large regions, thus needing a large number of sensor nodes. In order to reduce the cost of these nodes, it is customary to use low-cost analogue sensors along with a programmable electronic interface capable of adapting every sensor output to the port requirements of the microcontroller (μC) embedded in the sensing node. Such a reprogrammable sensor interface widens the range of applications and thus eases the marketability of the interface circuit sensing solution. In the literature, implementations of such systems have been recently reported, e.g., designed for gas sensor arrays conditioning [2] or industrial environments [3]. In [4] a portable general programmable sensor interface is presented based on a commercial System on Chip (SoC). This system allows connecting several sensor types, including sensors with digital output and smart sensors and provides several standard communication protocols. To allow all these capabilities, the programming interface becomes complex, thus requiring a specific development environment and some programming background, while plug and play capability is achieved through specific detection and trigger lines, increasing the required input and output resources of the interface-to-master module bus where the system is connected.

The goal of the present work was the design and experimental validation of a simple plug & play programmable sensor-to-μC interface able to self-configure its operation when adapting the output of different sensors, optimizing every sensor span. The proposed Smart Transducer Interface Module (STIM) includes both electronic and software elements. The hardware module consists of an electronic system that transforms the output of resistive sensors and sensors with voltage/current output to a quasi-digital signal compatible with the electrical levels of the digital input ports of the low-power μC in the sensor node, thus allowing easy reading [5]. This electronic interface system can be reprogrammed according to the electrical characteristics of the connected sensor so as to achieve an optimum sensor reading performance. This is done by the software module, implemented into a small auxiliary μC which adjusts the programmable electronics to optimize the conditioning circuit operation and coordinates the measurement process managing the resources involved in the operation. The information to properly configure the hardware module and recover the value of the measured magnitude from the sensor reading is contained in a small flash memory in this auxiliary μC. In addition, the proposed interface is plug & play (P&P), containing all the required information for configuration when it is plugged into the master μC of the sensor node, self-configuring its operation without user interaction.

The paper is organized as follows. Section 2 describes the proposed smart transducer interface design. Section 3 explains the software design for the conditioning and communications processes, including the final frequency to code conversion performed in the master μC. Section 4 shows the system implementation and analyses power consumption in a wireless sensor node. Section 5 presents the application of the proposed system as an interface for some low-voltage sensors, in particular for a temperature dependent resistor (NTC), a humidity dependent resistor (RH sensor), a linear Hall sensor, a light dependent resistor (LDR) and a photodiode. Finally, conclusions are drawn in Section 6.

## 2. STIM Electronic Interface

The proposed sensor interface can accommodate resistive sensors and sensors with voltage/current output. In addition, it is compatible with the needs and restrictions of the nodes of a wireless sensor network: low-voltage, to be powered with low form factor batteries; minimum power consumption, to optimize the node life; and low-cost, to minimize the total cost for WSN applications involving a high number of nodes spread out over large areas.

**Figure 1.**
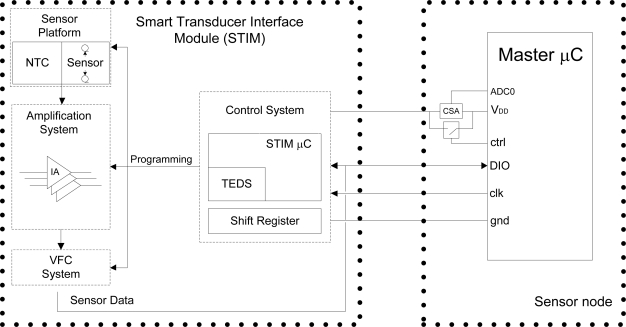
Complete scheme diagram of the proposed sensor interface and communications.

A simplified diagram of the interface circuit is shown in Figure 1. The different output voltage ranges provided by the different sensors connected to the sensor platform are converted into a common voltage span by means of the amplification system block, which digitally adjusts its gain and offset voltage depending on the sensor signal characteristics. This common voltage span is next converted through a voltage-to-frequency circuit (VFC) into a pulse signal whose frequency proportionally depends on the input voltage. Frequency conversion is selected because frequency-coded information shows much less sensitivity to interference [5]. Furthermore, to achieve the best performance in the subsequent frequency conversion to digital values, the sensor common voltage span must cover the complete 0-V_DD_ supply voltage. The quasi-digital signal provided by the VFC is read directly by the sensor node master μC using a single digital input/output port. The master μC then digitizes the data using the Direct Counting Method (DCM) [6] and transfers the results to the sensor network coordinator by a wireless protocol.

The STIM μC is responsible for scheduling the three blocks of the proposed sensor interface: Sensor Platform, Amplification System and VFC System. In addition, the plug & play concept is used to self-configure this interface when it is connected to a sensor node microcontroller. Configuration data are stored in a small memory in the STIM μC similar to TEDS (Transducer Electronic Data Sheet) sensors [7]. Communication between the STIM interface and the sensor node is carried out through two lines: a clock line (clk) and a bidirectional data line (DIO).

### 2.1. Sensor Platform

**Figure 2.**
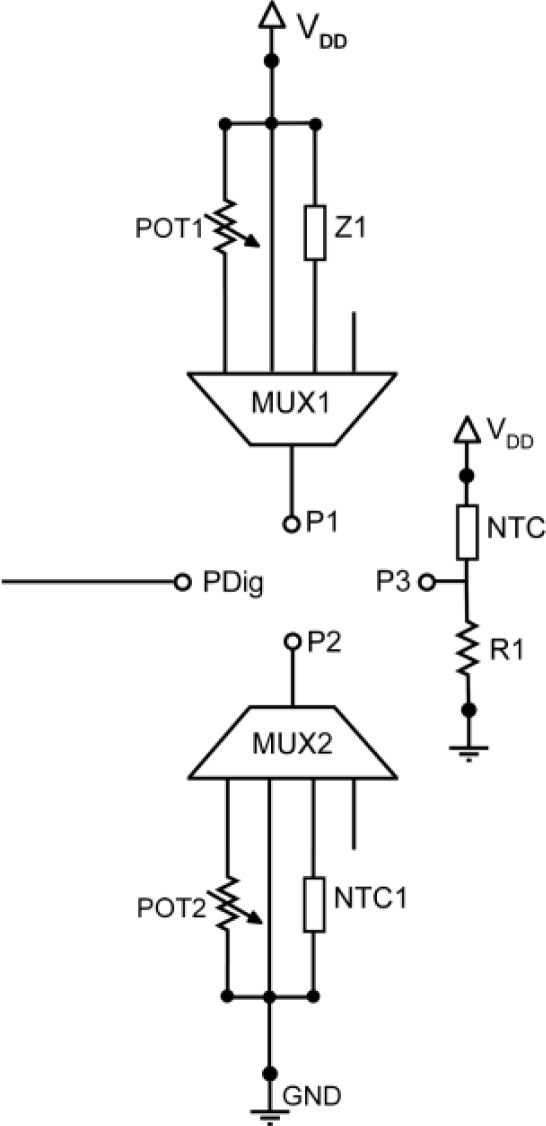
Sensor platform.

Figure 2 shows a schematic of the sensor platform. It includes two pins to connect the sensors and basic reconfigurable conditioning electronics to adequately transform changes in voltage, current or resistance to voltage variations that will then be amplified to fit the full output voltage span. Thus, each sensor is connected between terminals P1 and P2 driven by 4:1 analog multiplexers MUX1-MUX2, which allow setting the suitable basic conditioning scheme for each sensor type.

Resistive sensors R_SENSE_ employ a resistive divider as basic conditioning electronics. As shown in Figure 2, if R_SENSE_ is connected between terminals P1 and P2, a resistive divider POT1-R_SENSE_ or, alternatively, R_SENSE_-POT2 can be formed between V_DD_ and gnd properly configuring MUX1 and MUX2. POT1 and POT2 are programmable resistances implemented by linear digitally programmable potentiometers, whose value is adjusted depending on the sensor characteristics. In addition, the platform includes a grounded Negative Temperature Coefficient (NTC) resistor NTC1 to adequately condition a low-cost resistive humidity RH sensor. This thermistor is used to self-compensate the RH temperature output drift. Impedance Z1 is actually a socket, so it can be replaced with any particular resistance or component according to the specific conditioning characteristics of other sensors used in the platform.

I_SENSE_ current output sensors are usually conditioned employing a resistor in series which converts the current into voltage. Typically these sensors need to be fed and their basic conditioning scheme is similar to those of resistive dividers. Then, with I_SENSE_ connected between P1 and P2, MUX1-MUX2 will be configured to form the series connection I_SENSE_-POT2 between V_DD_ and gnd. In this way, V_DD_ supplies the current sensor, and current variations are transformed, through an adequate value of POT2, to voltage to be further processed. Voltage output sensors V_SENSE_ do not require a specific conditioning step, but usually need to be fed. So, V_SENSE_ will be connected between P1 and P2 and these pins directly through the MUX1-MUX2 lines to V_DD_ and gnd. Voltage differential sensors are directly connected between the unconnected MUX1-MUX2 lines, so that the voltage difference is amplified in the next stage with the use of differential instrumentation amplifiers. To properly perform the differential amplification, the positive terminal is connected to P1 and the negative to P2.

Since many commercial sensors are greatly dependent on temperature, a NTC sensor has been included within to check the internal temperature and apply thermal compensation, so that accurate readings of the physical quantities can be taken [8]. A low-cost NTC is chosen as a temperature sensor as it has a known calibration curve and requires as a conditioning circuit a simple resistive divider, as shown in Figure 2. Its output pin P3 is directly connected to the amplification system. Finally, PDig pin is a digital output to drive the control input of some commercial analog sensors in order to enable low-power modes.

To implement this sensor platform, the selected POT1 and POT2 potentiometers are MAX-5414 from Maxim, with a nominal value of 50 kΩ and 256 taps or digitally controlled different positions for their mobile terminal [9]. Analog multiplexers MUX1 and MUX2 are ADG704 (4:1) from Analog Devices [10], NTC and NTC1 are 4K7 resistors at 25 °C from Vishay [11] and R1 = 2.7 kΩ.

### 2.2. Amplification System

**Figure 3.**
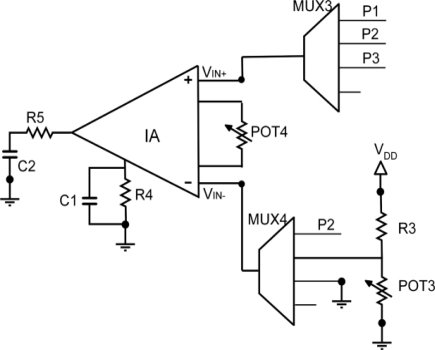
Amplification System.

Figure 3 shows the schematic of the proposed amplification block. It mainly consists of a programmable voltage adapter circuit that performs the subtraction of an offset voltage and the multiplication of the signal by a programmed value, to convert the different sensor output ranges to a common voltage range from 0 to 3 V, corresponding to the sensor node supply voltage. So, the gain and offset voltage used to suit the sensor signals allow obtaining maximum voltage resolution.

The circuit basically contains an instrumentation amplifier IA and two programmable potentiometers POT3 and POT4. The sensor output voltage V_SENSE_ can be driven from P1 or P2 to the non-inverting IA input V_IN+_ through the 4:1 multiplexer MUX3. An offset signal V_offset_, generated through the R3-POT3 resistive divider, can be driven to the inverting input V_IN−_ through MUX4.

The voltage at the output of the IA is proportional to the difference of the voltages at its inputs and the ratio of resistors R4 and POT4, as described by the following equation:

(1) $$V_{\mathrm{OUT}}=2\frac{R_{4}}{{\mathrm{POT}}_{4}}(V_{\mathrm{IN}+}-V_{\mathrm{IN}-})$$

By properly connecting V_SENSE_ to V_IN+_ and V_offset_ to V_IN−_ the IA output voltage is given by

(2) $$V_{\mathrm{OUT}}=2\frac{R_{4}}{{\mathrm{POT}}_{4}}(V_{\mathrm{SENSE}}-V_{\mathrm{offset}})$$

Therefore, the amplifier gain can be digitally controlled through the value of POT4, configured as a variable resistor.

According to Equation (2), the subtraction of the offset voltage is performed before the amplification. This offset voltage is given by the simple equation of a voltage divider made up of R3 and POT3:

(3) $$V_{\mathrm{offset}}=V_{\mathrm{DD}}\frac{{\mathrm{POT}}_{3}}{{\mathrm{POT}}_{3}+R_{3}}$$

The value of R3 equals the nominal value of potentiometer POT3 to achieve with high resolution a range of appropriate offset values from, approximately, 0 to V_DD_/2. The appropriate resolution of the voltage values would not be possible to achieve considering only the potentiometer POT3 working as a resistive divider between V_DD_ and gnd due to the coarse voltage discretization in the potentiometer output associated to the maximum number of programmable levels (256) available. Note that if the subtraction of an offset to the sensor signal is not necessary, MUX4 allows the grounding of the IA input V_IN−_; to make this possible the IA amplifier must also be rail-to rail at its input.

In the case of differential amplification, the positive sensor output V_SENSE+_ is driven from P1 to the non-inverting IA input V_IN+_ through MUX3, and the negative sensor output V_SENSE−_ is driven from P2 to the inverting IA input V_IN−_ through MUX4. The IA output voltage is then given by:

(4) $$V_{\mathrm{OUT}}=2\frac{R_{4}}{{\mathrm{POT}}_{4}}(V_{\mathrm{SENSE}+}-V_{\mathrm{SENSE}-})$$

which, as already said, can be digitally controlled through the value of POT4.

The instrumentation amplifier used in the amplification block is a rail-to-rail input and output INA327 from Texas Instruments [12]; MUX3 and MUX4 are ADG704 (4:1) from Analog Devices [10]; and potentiometers POT3 and POT4 are MAX-5415 from Maxim [9], with a nominal value of 100 kΩ and 256 taps, so this very number of possible gains and offset values can adjust the sensor characteristic. The RC circuit (R = 100 Ω, C = 1 μF) located at the IA output filters the signal, as recommended in the amplifier datasheet [12].

### 2.3. Voltage-Frequency Conversion System

The output of the previous amplifier block is a voltage signal V_OUT_ whose value ranges from 0 to 3 V. The next conditioning block consists of a voltage-controlled oscillator (VFC) which performs the transformation to the frequency domain, according to the expression:

(5) $$F_{\mathrm{OUT}}=0.1\cdot f_{\mathrm{clk}}+0.8\cdot \frac{V_{\mathrm{OUT}}}{V_{\mathrm{DD}}}\cdot f_{\mathrm{clk}}$$

where *f_clk_* is a reference clock frequency provided by the STIM μC to the VFC and set to 500 kHz, and V_DD_ is the supply voltage, 3 V in our case. Therefore, the output frequency ranges from 50 kHz to 450 kHz, which are adequate values to be afterward processed in the master μC driven at 4 MHz. In addition, the VFC output signal is fully compatible with the logic levels of the digital input/output ports of the master μC. Therefore, the VFC data output signal is driven directly to a digital I/O port of the master μC, which performs the digitalization using the DCM [6]. Results are then transferred to the sensor network coordinator. The selected commercial VFC is an AD7740 from Analog Devices [13]. This is the only commercial VFC found by the authors that complies with the supply requirements (3 V) of WSN applications.

### 2.4. Control System

The control block is responsible for scheduling, activating and configuring all the interface electronics. It consists of a microcontroller and an 8-bit shift register. The selected STIM μC is an MC9RS08KA8 from Freescale [14]. It is a 20-pin low-cost μC, specially designed for low power applications. It handles the communications with the sensor node master μC, based on the I2C protocol, and manages the interface electronics. This device allows the implementation of plug & play technology, using Transducer Electronic Data Sheets (TEDS) in a similar way to the IEEE 1451.4 standard [7]. This is achieved thanks to its small memory unit containing the information about the different sensor characteristics necessary for the plug & play functionality, such as operation ranges, conditioning parameters, resistors values, *etc*.

The STIM μC has two different data storage elements: a volatile random access memory (RAM, 256 bytes, fast access) and a non-volatile Flash memory (8 Kbytes, non-volatile and slower than RAM). The STIM μC stores all the electronic datasheets of the connectable sensors in the non-volatile flash memory, thus avoiding being deleted if the microcontroller is turned off. At the STIM power-up time, the connected sensor is recognized and its datasheet, preceded by the NTC datasheet which is included to perform temperature calibration, are mapped into the RAM memory to allow a faster access at runtime and not slow down the process. This datasheet information is then used to properly configure the electronic interface of the NTC, followed by that of the sensor.

**Figure 4.**
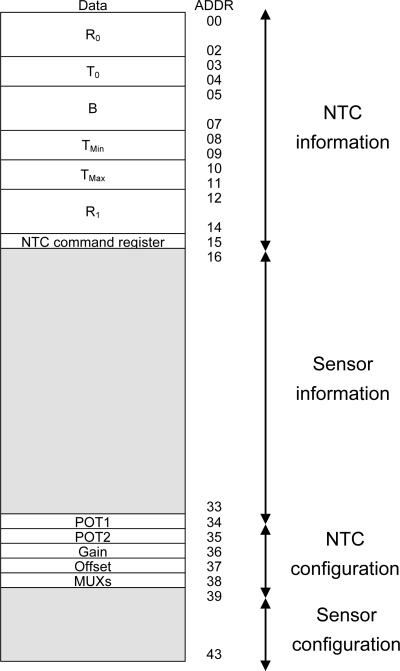
STIM μC’s RAM memory with NTC information.

Figure 4 shows the structure of the STIM RAM memory where the information is saved. The first 16 bytes correspond to the NTC TEDS, and the following 18 bytes to the sensor TEDS. The latter number, suitable for all the sensors considered here, can be increased or decreased according to the characteristics of the sensors to be used. The last TEDS position (15 for the NTC and 33 for the sensor) are reserved to store the commands sent by the master μC to the STIM. TEDS, positions 34 to 38 then store the settings for conditioning and performing a reading of the NTC. Positions 39 to 43 store these same parameters for the corresponding connected sensor. Figure 4 details the specific information stored in each register block—information and configuration—for the NTC.

The 8-bit shift register, a Texas Instruments SN74HC594D [15], contains appropriate control signals for all MUX1 to MUX4 multiplexers. Its programming is performed as follows: the STIM μC writes in series the 8-bit control values on the register; once the register contains the eight suitable control signals to set the paths of the multiplexers, the STIM μC uses the I/O available resources to: (1) control the fed switch (1 I/O port); (2) program the digital potentiometers (three I/O ports); (3) enable the multiplexers and the instrumentation amplifier and control the PDig pin (3 I/O ports); (4) create the VFC frequency reference signal (1 I/O port); (5) communications with the sensor node (2 I/O ports) and (6) drive the shift register; gnd, V_DD_ and two ports reserved to program the microcontroller complete the 17 available I/O STIM μC ports. Therefore, the 8-bit shift register enables to virtually increase the number of microcontroller I/O pin number by performing a series-parallel conversion.

### 2.5. Plug & Play Implementation

Plug and play is a feature in which both the device to be connected as well as the host device must show some specific characteristics. To allow this functionality, the host system needs to be able to detect the connection at one of its ports of the P&P device in order to start the identification and configuration protocol; conversely, the connected device needs to include the required information and be able to send it to the host device when requested. Although this protocol can be fully software-implemented, this increases the requirements of the STIM μC, as well as its cost. Thus, a mixed hardware-software technique has been selected in this work.

#### 2.5.1. Interface Device

Figure 1 shows the four lines that connect the host device, *i.e*., the master μC, and the STIM: V_DD_, gnd, clock (clk) and a bidirectional data line (DIO). When a new STIM interface is connected to these lines, the STIM μC turns off the power line of the conditioning modules (Sensor Platform, Amplification System, VFC System), and drops to a low power mode. Under these conditions, the full one STIM interface current consumption is 4 μA. When the master μC wakes up and detects through changes in current consumption that a new interface has been connected, an I2C address request is sent. All the connected STIM microcontrollers wake up to verify the instruction, returning to a low power mode and only the new device sends its pre-defined address and the sensor information stored in the flash memory, completing the plug & play operation and returning to a low power state until a measurement process is requested. When a STIM is unplugged from the host device, the system detects a reduction in current consumption; the next time the host will address each one of the STIM interfaces only the connected devices will give an answer. The sensor node will delete from its database the I2C address of the disconnected device, thus updating the interface list. Note that when a connected interface is replaced by a different STIM, it is necessary to wait for two detection phases for a correct operation.

#### 2.5.2. Host Device

In order to detect a new hardware connection, the V_DD_ line that provides power to the STIM devices is monitored each time the sensor node is awakened, thus detecting any changes in the current flow. Current detection is performed by an LMP8645 precision Current Sense Amplifier (CSA) [16], as shown in Figure 1. The CSA is connected to the bias line through a 30 Ω shunt resistor. Thus, when a new interface is connected to the bus, the current in the bias line is increased 4 μA due to the quiescent current of the STIM μC in low power mode (the rest of the electronics remain unbiased as explained previously). With a 330 kΩ gain resistor placed in the output of the CSA to amplify the voltage across the shunt resistor, the increase in the bias line current results in a voltage rise of 8 mV for each connected STIM at the CSA output, with an initial offset of 15 mV, which is read by one of the ADC lines of the master μC. This ADC is a 10-bit converter with an offset of 2 bits, so it is able to detect the considered voltage increments and, hence, whether a new device is connected. Before the master μC sends the I2C address request and the connected STIMs wake up, increasing the current consumption, a switch in parallel with the CSA shorts the 30 Ω shunt resistor to avoid the voltage drop and provide a suitable V_DD_ (Figure 1). In order to minimize the effects of noise, the analog to digital conversion is performed by using an ADC noise reduction operation mode available in the host microcontroller. In addition, the STIM interface includes filtering capacitors in the supply lines. In the case of noisy environments, the shunt resistor can be increased to obtain a greater voltage in the CSA output, thus reducing the system sensitivity to noise.

## 3. Software Design

After designing the hardware interface the STIM μC must be programmed to manage the whole electronics of the interface and the communications protocol with the WSN node, optimizing the resources to obtain an efficient implementation: in WSN systems, where power is provided by small form-factor batteries, it is a priority to minimize the power consumption to extend the operational battery life. Thus, in these applications the electronic system is set into a low-power mode most of the time, waking up to perform the interface programming, measurements, frequency to digital conversions and the final radiofrequency data transmission, then returning to the sleep mode where the power consumption is minimal.

**Figure 5.**
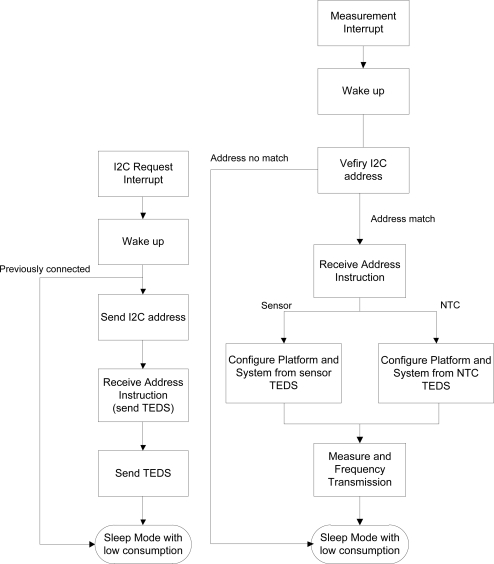
Basic STIM μC operations flow for (**left**) first connection and (**right**) measurement request.

Figure 5 presents the STIM μC operating diagram. When the interface is connected the STIM is taken out from the sleep mode by a master μC I2C address request interrupt (communications between the STIM and node microcontrollers are based in the I2C protocol [17]) and sends the I2C address corresponding to the own STIM μC. Then, in the corresponding memory address, it receives the command to be executed (position 15 in RAM for the NTC and 33 for the sensor, Figure 4). The first time the STIM μC receives an interrupt, the request is to send the electronic datasheet of the connected sensor to the master μC. The following times the STIM μC receives an interrupt, the requested operation is to configure the interface and the output to perform a measurement process. Therefore, the control system properly selects the inputs and the corresponding gain and offset (interface configuration), selects the path lines to send the measured data as a frequency-coded signal (output configuration) and the STIM μC returns to the sleep mode (low power). At that moment, the master μC receives the sensor data for its digitalization, transferring the results to the sensor network coordinator by a wireless protocol. Once the data is digitized, the measurement information can be recovered from the TEDS information sent to the master μC.

The use of two different memory positions for commands storage in the STIM μC (one for the embedded NTC instructions and the other for the connected sensor instructions) makes possible to send two different commands to be executed in parallel. Although this is not currently implemented, the STIM for example could configure the conditioning electronics according to the sensor TEDS and send the output signal to the master μC, while it updates some of the TEDS information of the embedded NTC sensor according to a new calibration, thus minimizing the runtime.

**Figure 6.**
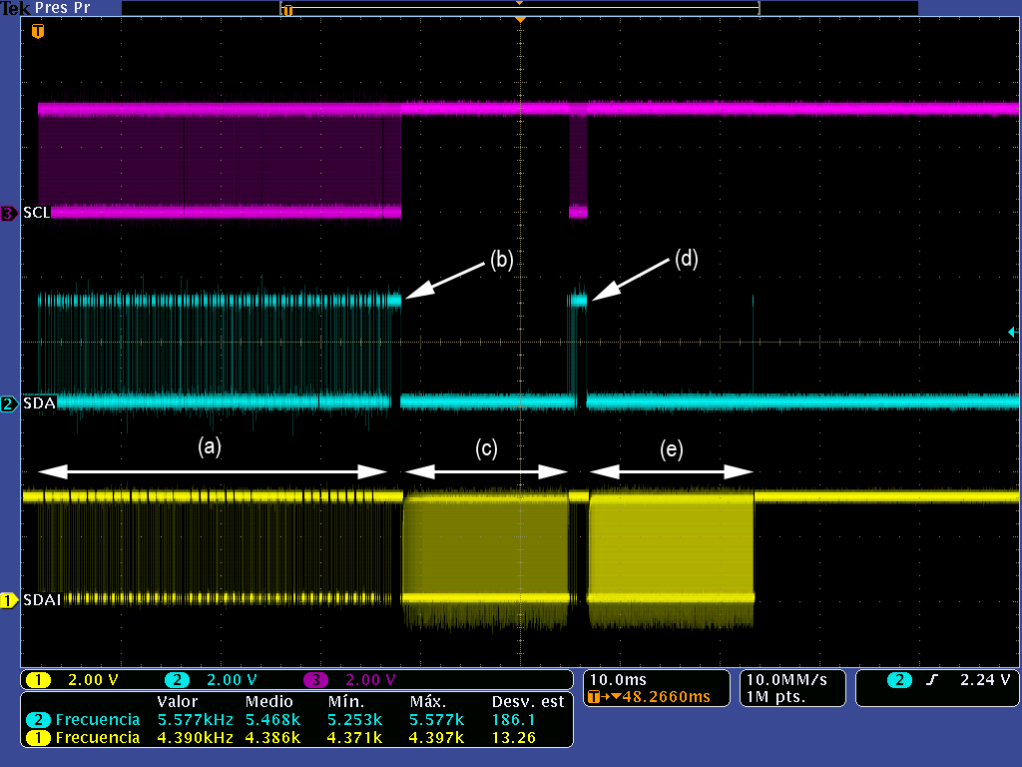
Chronogram of a first plug and measurement of the STIM into the sensor node.

Figure 6 shows a chronogram of a first connection of the proposed STIM to the sensor node after identification followed by a measurement process. For a better display of the data in the bidirectional DIO line we have split it into two different channels: SDA (from master μC to STIM) and SDAI (from STIM to master μC). Clock line (SCL) is only active for I2C communications between both systems. The process shown in the figure presents several steps: after the STIM is recognized by the master μC the interface sends the TEDS of the connected sensor to the node (a); the master μC then sends a Receive Address Instruction (b); the STIM interface is then fully configured, performing a measurement process for the NTC embedded in the interface (c); after that, another Receive Address Instruction (d) configures the STIM to perform a measurement of the connected sensor (e). Once the process is finished, the STIM returns to the low-power mode, waiting for the next interrupt to start the measurement process again.

**Figure 7.**
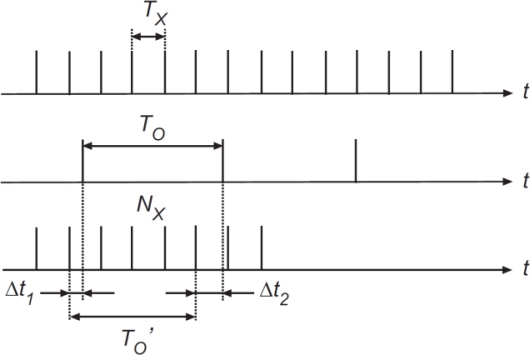
Direct Counting Method timing diagram.

The frequency to digital value conversion is performed in the master μC using the classical Direct Counting Method (DCM) [6]. This method counts the number of pulses (*N_x_*) of a signal of unknown period (*T_X_*) in a temporal window defined by *n* periods of a signal of known frequency (*f_0_*). Figure 7 shows the timing diagram of the DCM.

The number of pulses into the counting window is given by:

(6) $$N_{X}=n\cdot \frac{T_{0}}{T_{X}}$$

where *T_0_* is the period of the known signal. The unknown frequency *f_x_* is calculated by the number of pulses into the counting window:

(7) $$f_{x}=\frac{N_{x}}{n\cdot T_{0}}$$

The bigger *n* is, the greater the accuracy, but at the cost of a longer calculus time. In our case, using a signal of period *T_0_* = 2.5 × 10^−7^ s (4 MHz), *n* = 65,535 provides a suitable tradeoff between accuracy and operation time, giving an accuracy higher than 12 bits for measuring times of 16 ms.

Thus, the master μC in the sensor node receives the frequency coded value and translates it into a digital value according to the DCM. Before the data are sent by the sensor node to the WSN coordinator, the recovered value is converted to the measured value of the physical magnitude by properly applying the information stored in the corresponding TEDS, previously sent to the master μC in its first connection (Figure 5(left)).

## 4. System Implementation and Power Consumption Considerations

**Figure 8.**
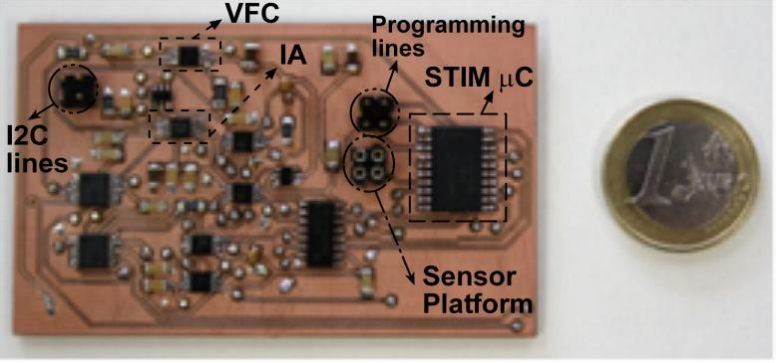
Photograph of the sensor interface (dimensions: 76 × 46 mm).

The designed circuit has been implemented using Commercial Off The Shelf (COTS) components. Since it targets an application using battery-operated WSN nodes, it must have reduced power consumption and, accordingly, at the circuit level, the components used for its implementation must be compliant with the Low-Power Low-Voltage (LPLV) requirements. The selected components are summarized in Table 1. Note that all these components have low-power modes to allow a selective device enabling in order to optimize the operating power consumption, except the VFC AD7740 [13] whose typical power is 3 mW and no low-power mode is available; so a switch connects and disconnects this device from the power supply to implement a low-power VFC mode. Figure 8 shows a photograph of the proposed system.

At the system level, to minimize the power consumption, the interface should be active only when the node requests a configuration and measurement process and remain off the rest of the time. Therefore an ADG701 switch from Analog Devices [18] controlled by the STIM μC is used to connect and disconnect the power supply from the whole STIM electronics. Thus, in sleep mode the conditioning electronics (Sensor Platform, Amplification System, VFC System) remain unbiased and the microcontroller is diverted to a low power mode. When the STIM interface is in the configuration and measurement steps, the microcontroller is awake and the power line is connected. By means of the corresponding enable terminals of the components and suitable switches it is possible to power on only the required electronics at each step, thereby reducing the power consumption.

**Figure 9.**
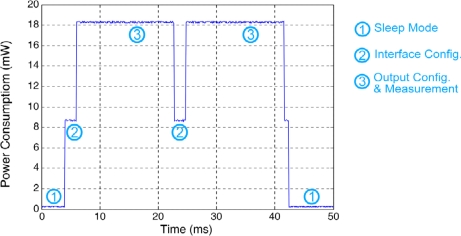
STIM power levels.

Figure 9 shows the consumption levels of the STIM interface, tested in a WSN mote that complies with the IEEE 802.15.4 standard [19]. It consists of an XBee transceiver from Digi and an Atmega1281 from Atmel, powered by two 1.5 V–1,500 mAh LR06 batteries [20]. Over a complete measurement cycle and depending on the interface state, the system presents different power consumption values: the lowest level, below 12 μW, corresponds to the sleep mode, *i.e*., the STIM μC is in low power mode and the whole STIM electronics are turned off using the mentioned power switch. The second power level (8.7 mW, 2 ms) corresponds to the embedded NTC interface configuration step, giving the suitable values to the potentiometers (POT1, POT2, gain, offset) and the multiplexers. The third level (18.3 mW, 16.8 ms) corresponds to the NTC output configuration and measurement, biasing the sensor and enabling the multiplexers, the IA and VFC System. The next two power consumption levels (8.7 mW, 2 ms; and 18 mW, 16.8 ms) are again interface configuration and output configuration and measurement levels, but for the specific connected sensor, which in the case of Figure 9 is again the NTC. Power consumption level 2 remains unchanged for every sensor, but this last power consumption (18 mW) can increase or decrease depending on the connected sensor power requirements. Data are transmitted to the sensor node μC while the measurement process is performed by the STIM controller. After the measurement process is complete, the system returns to the initial low power state.

## 5. Application Examples

The interface is compatible with most of the common types of sensors used in WSN applications. For this work, the sensors have been selected so as to be LPLV compliant. Another important characteristic is the sensor cost: in these applications, where a large number of nodes will be spread over large areas, low-cost is a prerequisite.

The selected resistive sensors are an NSL-19M51 LDR (Light Dependent Resistor) from Silonex [21], a H25K5A humidity sensor from Sencera [22] and an NTC [11]. The NTC undergoes relatively small changes in the value of their resistance, while the LDR and the humidity sensor present a high output span. As a voltage output sensor an A1391 linear Hall-effect sensor from Allegro [23] has been selected. The chosen current output sensor is an S8265 photodiode from Hamamatsu Photonics [24], whose output variations are very small.

Each sensor has its own electronic datasheet where variables such as model parameters or operating ranges are stored. Once the sensor application is selected, the maximum V_MAX_ and minimum V_MIN_ sensor output is known. So, the gain and the offset can be calculated according to the following equations:

(8) $$\mathrm{offset}=V_{\mathrm{MIN}}$$

(9) $$\mathrm{gain}=\frac{V_{\mathrm{DD}}}{V_{\mathrm{MAX}}-V_{\mathrm{MIN}}}$$

To show the performance of the proposed system, its operation when applied to conditioning the aforementioned NTC, a RH sensor, a LRD sensor, a linear Hall sensor and a photodiode is as follows.

### 5.1. NTC

NTC is a resistive sensor R_SENSE_ whose resistance value depends on temperature T_SENSE_, reducing its value with any increase in temperature. Its dependence is exponential, according to the equation:

(10) $$R_{\mathrm{SENSE}}=R_{0}\cdot e^{B\left(\frac{1}{T_{\mathrm{SENSE}}}-\frac{1}{T_{0}}\right)}$$

where B is a characteristic constant and R_0_ is the sensor resistance value at a known temperature T_0_, usually 298 degrees Kelvin. For this work, both values were experimentally obtained from a complete NTC characterization, resulting B = 4,007 K and R_0_ = 4,705 Ω at 278 K.

The NTC conditioning circuit is a resistive divider R_SENSE_−R_1_ between V_DD_ and GND, so that resistance variations are converted to voltage variations:

(11) $$V_{\mathrm{OUT}}=V_{\mathrm{DD}}\frac{R_{\mathrm{SENSE}}}{R_{\mathrm{SENSE}}+R_{1}}$$

The R_1_ value is calculated according to the conventional criteria of having a maximally linear response within the temperature operating range, as given by the following expression:

(12) $$R_{1}=R_{T_{C}}\frac{B-2T_{C}}{B+2T_{C}}$$

where T_C_ is the operating range central temperature and R_TC_ is the value of R_SENSE_ at T_C_. For the typical range of temperature measurement in environmental applications [−20 °C, 80 °C], T_C_ = 30 °C and thus the value of R_1_ is about 2.7 kΩ. All these values (R_0_, T_0_, B, T_MIN_, T_MAX_, R_1_) are stored in binary format in the NTC TEDS, in conjunction with the binary words to properly configure the conditioning and reading electronics of the STIM. Figure 4 shows the corresponding memory map and Table 2 summarizes all these values.

**Figure 10.**
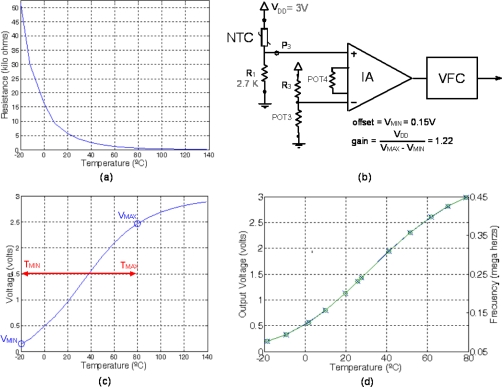
NTC behavior: (**a**) Experimental characterization; (**b**) Conditioning interface scheme; (**c**) Resistive divider output voltage, function of the corresponding sensed temperature; and (**d**) Conditioned output sensor range fit to a common 0–V_DD_ range (x) and VFC output (o).

Figure 10(a) shows the NTC experimental characterization. Figure 10(b) depicts the block diagram of the proposed interface applied to the NTC sensor at temperatures ranging from −20 to 80 °C. The NTC-R_1_ resistive divider provides the output voltage function of the corresponding sensed temperature, as shown in Figure 10(c). The output is driven to the IA, programmed to reduce the offset and vary the gain so that, within our [−20 °C, 80 °C] operating range, the output voltage signal spans the entire range (0–3 V) of the supply voltage, optimizing the input range to the VFC and maximizing the system sensitivity. Figure 10(d) shows the 0–3 V output voltage read by the master μC and the output frequency also recovered in the master μC.

### 5.2. Humidity Sensor

The H25K5A humidity sensor used in this work is a resistive element whose resistivity depends on both the relative humidity and the temperature according to a complex expression. Thus, the sensor operation is not usually given by an equation, but rather by a table in which the sensor output value is provided for different ambient humidity and temperature values. However, the table size is too big to be stored in the sensor node memory. Thus, the final network processing system (usually a computer) that receives all the WSN information from a coordinator node must keep it in a file describing this sensor behavior and, to properly process the output signal, the name of this file is stored in its corresponding electronic datasheet and sent to the master μC in the first STIM connection process. Then, the first time the node is connected to the network coordinator it sends the name of the file. By using the corresponding data file, the network processing unit is able to recover the measured RH value from the temperature and R_SENSE_ values sent by the corresponding STIM.

**Figure 11.**
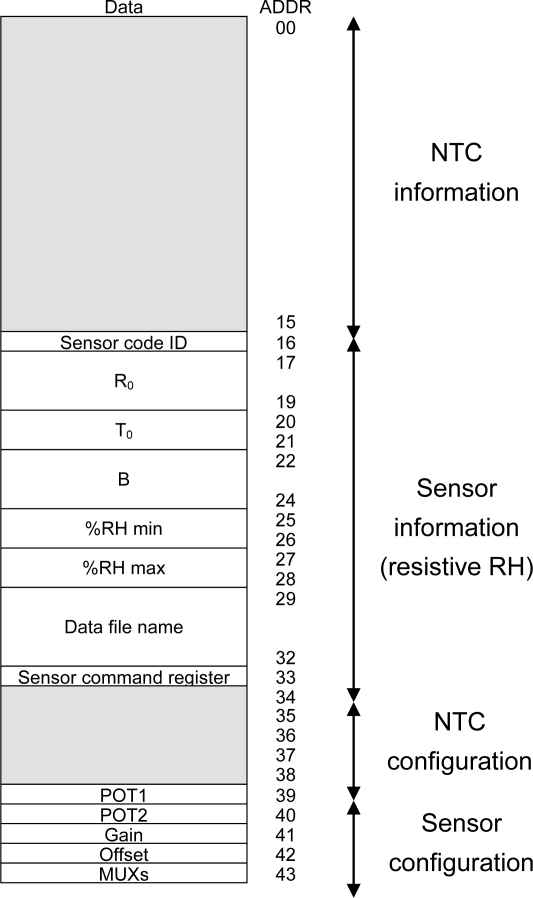
STIM μC’s RAM memory with RH sensor information.

The conditioning circuit for this sensor is a resistive divider R_SENSE_-NTC1 between V_DD_ and gnd, while the temperature value is known thanks to the NTC embedded into the interface. So, once the value of the sensor resistance is calculated and the temperature value is known, the table gives the RH estimation. Figure 11 shows the memory map of the parameters stored in the TEDS for this sensor and Table 2 shows the stored values. It includes the parameters of NTC1, the sensor RH operating range and the name of the characteristic data file.

**Figure 12.**
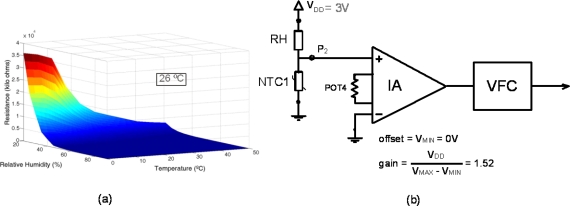

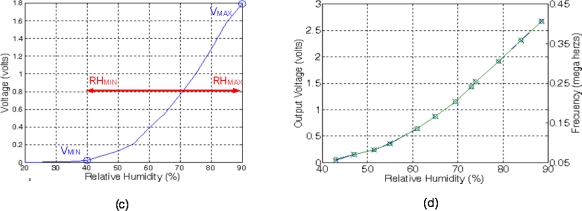
Humidity sensor behavior at 26 °C: (**a**) Experimental characterization; (**b**) Conditioning interface scheme, (**c**) Resistive divider output voltage, function of the corresponding sensed temperature; and (**d**) Conditioned output sensor range fit to a common 0–V_DD_ range (x) and VFC output (o).

Figure 12 shows, similarly to the NTC example, the results of applying the proposed interface to this sensor at 26 °C for relative humidity ranging from 40 to 90%.

### 5.3. Light Dependent Resistor Sensor (LDR)

**Figure 13.**
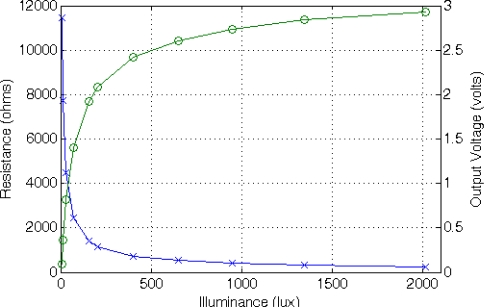
LDR behavior (x) and output voltage obtained from the master μC after the application of the DCM to the frequency-coded signal provided by the STIM (o).

The third sensor connected to the proposed interface to test its performance is a light dependent resistor NSL-19M51 from Silonex. Its resistance-to-light dependence is exponential, according to:

(13) $$R_{\mathrm{LDR}}=A\cdot L^{-\alpha }$$

where constants A and α, experimentally established, are A = 43,783 Ω·lux^α^ and α = 0.683. The basic conditioning circuit for an LDR is a resistive divider R_SENSE_-POT2 connected between VCC and gnd, similar to the circuit used to condition the NTC resistor, with a resistance value of 2.7 kΩ programmed in POT2. From these values, the proper TEDS field values are determined for a suitable sensor conditioning, acquisition and measure recovery. Figure 13 shows the LDR value as a function of the incident light ranging from 10 to 2,000 lux, and the conditioned output voltage recovered by the master microcontroller.

### 5.4. Linear Hall Sensor

The application of the proposed interface to a voltage-based sensor has been tested using an A1391 linear Hall sensor from Allegro. This sensor has been designed to deliver a voltage proportional to the distance to a magnet in a range of some centimeters, providing 5–10 μm accuracy. The input-output characteristic is given by:

(14) V=A⋅d+B

**Figure 14.**
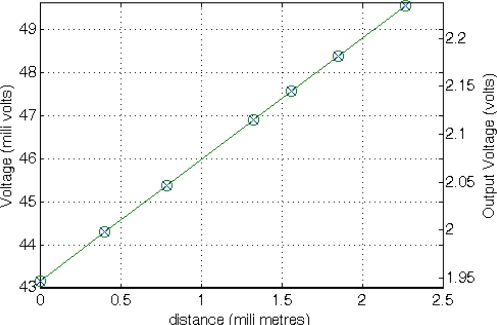
Hall sensor behavior (x) and output voltage obtained from the master μC after the application of the DCM to the frequency-coded signal provided by the STIM (o).

Coefficients A and B are obtained experimentally. In this case, A = 2.8187 V/m and B = 0.0417 V. Since this sensor is active, it must be connected to V_DD_ and gnd to be properly biased. In addition, it presents and additional low-power mode selection pin that is connected to PDig (Figure 2) to reduce energy consumption. The sensor output can be directly connected to the amplification system without additional resistors. We have configured the sensor platform to measure sensor displacements up to 2 mm with 10 μm accuracy. Figure 14 shows the sensor behavior and the voltage recovered by the master μC as a function of the distance to a permanent magnet in a 2.2 mm span.

### 5.5. Photodiode Sensor

Photodiodes are light sensors that have a faster response than LDRs to incident light variations making them suitable for use in systems where fast response times are required. Their output current depends on the incident light according to:

(15) $$L=A\cdot I_{0}+B$$

**Figure 15.**
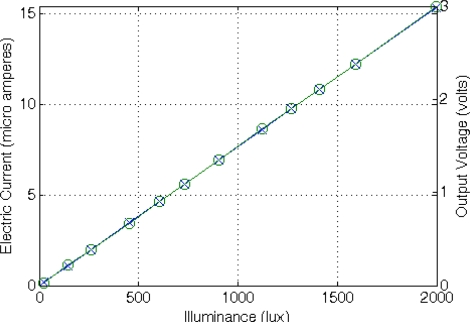
Photodiode output (x) and output voltage obtained from the master μC after the application of the DCM to the frequency-coded signal provided by the STIM (o).

Coefficients A and B were obtained experimentally: A = 130 lux/mA and B = 2.8419 lux. In this case, the sensor is connected in series with potentiometer POT2 between V_DD_ and gnd. POT2 is configured to a resistive value of 39 kΩ. Figure 15 shows the photodiode behavior for incident light from 10 to 2,000 lux and the voltage recovered by the master μC.

Table 2 summarizes the information stored in the corresponding TEDS for all the sensors used in this work. Shaded values are used only for programming both the Sensor Platforms, Amplification and VFC Systems, and so are not sent to the master μC in the first plug process of the STIM.

## 6. Conclusions

This paper presents a programmable conditioning interface for low-cost sensors in WSN applications. The proposed STIM can be connected to a sensor node by means of a standard I2C protocol and stores a reduced Transducer Electronic Data Sheet of the connected sensor to properly self-configure the conditioning electronics, thereby improving the output span, increasing the sensor sensitivity and reducing the temperature drift in the sensor response. For easy integration in the sensor node, this STIM uses a plug & play technique, which allows connection with no previous operation.

The interface can be used with a wide variety of sensors, and has been validated for a set of sensors including resistive sensors and sensors with voltage and current output. It provides a value of the measured parameter coded as the frequency of a signal compatible with the logic levels of the master μC that manages the sensor node resources, offering high immunity to noise and signal interferences.

**Figure 16.**
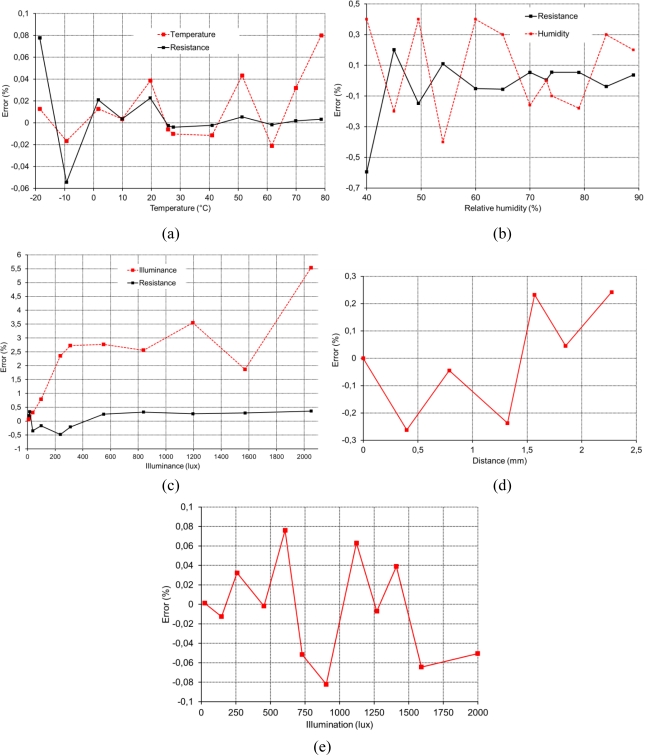
Full scale errors associated with the interface electronics for (**a**) NTC; (**b**) RH sensor; (**c**) LDR; (**d**) linear Hall sensor and (**e**) photodiode.

The frequency-to-digital value conversion can be easily performed in the master μC by means of the simple direct counting method, allowing more than 12-bit accuracy for conversion times below to 16 ms. Figure 16 shows the full scale error in the recovering of the sensor output magnitude compared to the value measured directly in the sensor output (*i.e*., the error due to the electronic interface). For those sensors without a linear relationship between the electric magnitude and the measured physical property (NTC, RH sensor and Hall), the correlated error for the corresponding physical property recovered from the sensor reading is also plotted. The obtained errors are compatible with the typical requirements of wireless sensor system applications, which handle with overall errors in the range of 1 to 5%.

By properly managing the interface electronics, the average power consumption in a measurement process of the conditioning electronics is compatible with the power requirements of portable sensing applications. Compared to previously proposed general purpose hardware interfaces based on the IEEE 1451.4 standard for intelligent sensors [4], the designed system is simpler and do not need to increase the required input/output resources of the interface-to-master module bus where the system is connected to implement the plug & play capability, thus leading to more efficient implementation.

## Figures and Tables

**Table 1.** Component characteristics.

	**Commercial Name**	**Single Supply**	**Temperature Range**	**Power Consumption**	**Other Characteristics**
MUX	ADG704	+1.8 V to +5.5 V	−40 °C to +85 °C	<0.01 uW	4:1 Rail-to-Rail Operation With Enable Pin
POT	MAX5414/5415	+2.7 V to +5.5 V	−40 °C to +85 °C	0.3 μW	50/100 kΩ Resistors Values 256 Taps Positions
IA	INA327	+2.7 V to +5.5 V	−40 °C to +125 °C	6.0 mW	True Rail-to-rail I/O Excellent Long-Term Stability With Enable Pin
VFC	AD7740	+3.0 V to +3.6 V or +4.75 V to +5.25 V	−40 °C to +105 °C	3.0 mW	Full-Scale Frequency Set by External System Clock
Shift Register	SN74HC594	+2.0 V to +6.0 V	−65 °C to +150 °C	0.2 mW	8-Bit Serial-In, Parallel-Out Shift Registers With Storage
Switch	ADG701	+1.8 V to +5.5 V	−40 °C to +85 °C	<0.01 μW	Rail-to-Rail Operation

**Table 2.** TEDS for the sensors applied in the work. Shaded values are not sent to the master μC.

**NTC parameters**		**%RH parameters**		**LDR parameters**		**Hall parameters**		**Photodiode parameters**	
R_0_	4,705 Ω	Type	1	Type	2	Type	3	Type	4
T_0_	25 °C	R_0_	4,730 Ω	A	43,783 Ω·lux^α^	A	2.8187 V/m	A	130 lux/mA
B	4007	T_0_	25 °C	α	0.683	B	0.0417 V	B	2.8419 luxes
T_MIN_	−20 °C	B	4004	Lux_MIN_	10	X_MIN_	0.0178	Lux_MIN_	10
T_MAX_	80 °C	%_MIN_	40%	Lux_MAX_	2,000	X_MAX_	0.0192	Lux_MAX_	2,000
R_S_	2,700 Ω	%_MAX_	90%						
		File	*Hume*						
POT1	0 kΩ	POT1	0 kΩ	POT1	0 kΩ	POT1	0 kΩ	POT1	0 kΩ
POT2	0 kΩ	POT2	0 kΩ	POT2	2.7 kΩ	POT2	0 kΩ	POT2	39 kΩ
Gain	1.22	Gain	1.52	Gain	1.30	Gain	45.1	Gain	5.0
Offset	0.15	Offset	0	Offset	0.50	Offset	0	Offset	0
MUX’s	0 × 05	MUX’s	0 × 6 B	MUX’s	0 × 79	MUX’s	0 × 49	MUX’s	0 × 7 B
